# Health insurance enrollment and vision health in rural China: an epidemiological survey

**DOI:** 10.1186/s12913-021-06754-0

**Published:** 2021-07-31

**Authors:** Xiaochang Yan, Baoqun Yao, Xi Chen, Shaoye Bo, Xuezheng Qin, Hua Yan

**Affiliations:** 1 National School of Development, Peking University, 100871 Beijing, China; 2 Department of Ophthalmology, Tianjin Medical University General Hospital, 300052 Tianjin, China; 3 Department of Occupational and Environmental Health, School of Public Health, Tianjin Key Laboratory of Environment, Nutrition, and Public Health, Center for International Collaborative Research on Environment, Nutrition and Public Health, Tianjin Medical University, 300070 Tianjin, China; 4 China Foundation for Disabled Persons, Dongcheng District, 100006 Beijing, China; 5 School of Economics, Peking University, 100871 Beijing, China; 6 Institute for Global Health and Development, Peking University, Beijing, China

**Keywords:** Health insurance enrollment, Vision health, Epidemiological survey, Rural China

## Abstract

**Background:**

Vision health is an important aspect of health worldwide. Visual impairment (VI) is associated with poor quality of life and is usually more prevalent in rural areas. To help rural populations obtain vision care, health insurance policies have emerged throughout the world. However, some existing literatures show that health insurance enrollment’s impact on the overall physical health of rural population has been minimal. Focusing on vision health among adults in rural China, our study aims to investigates the impact of health insurance on vision health, heterogeneity of the effect, and the moderating effect of health insurance enrollment on the impact of chronic physical diseases and basic eye diseases on vision health.

**Methods:**

Primary data were collected through a nation-wide epidemiological survey of vision health conducted in rural China in 2018, with a sample size of 28,787 used in our statistical analysis. Instrumental variables regression and Heckman selection models were conducted to examine the impact of health insurance enrollment and reimbursement ratio adults’ vision health outcomes. Subsample regressions by sex, age, education level, and whether with eye diseases were further conducted to explore the heterogeneity in our results. We then examined whether health insurance enrollment moderates the impact of chronic physical diseases and basic eye diseases on vision health through the method of introducing interaction terms.

**Results:**

Participating in health insurance reduced the probability of VI by 2.15 %. The reimbursement rate increasing by 1 % point may reduce the probability of worsening VI by 6.12 %. Men (-0.0235, *P* = 0.0002) benefit more from insurance enrollment than women (-0.0201, P = 0.0082) with respect to vision health. From the young adult group to the oldest group, the marginal effect of health insurance increased from − 0.0068 (*P* = 0.0394) to -0.0753 (*P* < 0.0001). The marginal effect on VI was most significant in people with lower education levels and weakened with increased education levels. People with basic eye diseases (-0.0496, *P* = 0.0033) benefit more from participating insurance than the people without basic eye diseases (-0.0196, *P* = 0.0001) with respect to vision health. The moderating effects of health insurance enrollment on the impacts of cerebral infarction (-0.1225, *P* < 0.0001), diabetes (-0.0398, *P* = 0.0245), hyperlipidemia (-0.1364, *P* = 0.0271), mental illness (-0.1873, *P* = 0.0010), glaucoma (-0.1369, *P* = 0.0073), diabetic retinopathy (-0.1560, *P* = 0.0043), and retinal vein obstruction (-0.2018, *P* = 0.0155) on vision health were significantly negative.

**Conclusions:**

The results suggest that participation in health insurance and higher health insurance reimbursement ratios reduced the risk of VI in the sampled adults. Health insurance has the most significant effect in in vulnerable groups. Heath insurance enrollment moderates the impacts of several chronic physical and basic eye conditions on vision health. Our findings have potential implications for reforming health insurance policies to improve vision health conditions in rural areas of developing countries.

**Supplementary Information:**

The online version contains supplementary material available at 10.1186/s12913-021-06754-0.

## Background

Vision health is increasingly recognized as an important outcome measure for health. As a common cause of disability and a major determinant to quality-adjusted life years [1], visual impairment (VI) is often closely related with a series of poor physical and psychological conditions [2], and is an important risk factor for many adverse events, including impaired physical function [3], mental health problems [4], reduced productivity at work and in daily life [5, 6], and decreased overall quality of life [7, 8].

VI is usually more prevalent in rural areas around the world [9]. First, disadvantaged by their lower socioeconomic status, rural populations are more likely to suffer from eye diseases than urban residents [10]. Second, because of insufficient health care resources in rural areas, rural populations often face accessibility barriers to vision care when suffering from eye diseases, which prevents them from getting effective treatment [11]. Third, limited medical knowledge may leave rural residents poorly informed about eye health and fail to realize the importance of timely medical treatment, and therefore be more likely to ignore a doctor’s advice or miss routine treatment, which may cause further deterioration of their health condition and irreversible vision damage [12, 13].

Considering these threats to vision health, health insurance policies have emerged throughout the world to help rural populations obtain vision care. Although policies differ by country, the common purpose is to reduce patients’ financial burden and out-of-pocket expenses. Empirical studies have evaluated whether these policies are effective in decreasing financial barriers to medical care and contributing positively to patients’ health. Some show that implementing health insurance policies in certain countries is effective. For example, not participating in vision insurance was demonstrated to induce higher risk of VI, reduce eye care utilization and cause irreversible vision damage in samples of US [14, 15], and Low- and Middle-Income Countries [16]. VI was also found to be associated with a lack of health insurance in samples of Korea, Trinidad, and Tobago [17, 18]. However, some people, especially the rural people, believe that the preventive care of health insurance is unnecessary, and they habitually obtain health services only when symptoms become severe [12]. A study based on a sample of US concludes that frequency of eye care utilization by people with health insurance was positively related to VI severity [19].

One reason for the seemingly contradictory results is that health insurance implementation is usually affected by confounding factors, such as healthcare utilization patterns among patients, physicians’ prescribing behaviors, etc. In general, health insurance policies should be designed to fit each country’s disease patterns and socioeconomic environment. In formulating specific policies, setting certain indicators, such as the policy’s standard minimum payment, reimbursement ratio, and maximum payment limit, are important for successful implementation, and will affect health outcomes in targeted population.

As a rapidly developing country, China is facing serious imbalance in the growth of its economic sector and its healthcare sector, and the reform of China’s health insurance system has undergone a long process and is still constantly evolving. In consideration of the affordability of medical services, China began to vigorously expand the health insurance coverage, as early as the late 1990 s. Initially, the Urban Employee Basic Medical Insurance (UEBMI) was established in 1998 for employees in the formal sectors. Subsequently, the New Cooperative Medical Scheme (NCMS) was established in 2003 for the rural residents, and the Urban Resident Basic Medical Insurance (URBMI) was established in 2007 for the urban poor and employees in the informal sectors [20]. Since the implementation of the three basic health insurance schemes, the number of people covered by health insurance has steadily increased in China. By the end of 2018, the number of people covered by basic medical insurance has reached 1,344.52 million (among them, 316.73 million people participated in the UEBMI, 897.41 million participated in the URBMI, and 130.38 million people participated in the NCMS), and the insurance coverage rate has remained above 95 % since then, meaning China has almost reached universal coverage by 2018. In recent years, the government has been committed to integrating the three basic insurance schemes to simplify the framework of the medical insurance system, to ensure that urban and rural residents enjoy equal access to health care and reimbursement services, and to improve the overall service efficiency of the medical insurance system. In January 2016, the State Council issued the “Opinions on the Integration of the Basic Medical Insurance System for Urban and Rural Residents”, aiming to merge the URBMI and NCMS into a unified Urban and Rural Resident Basic Medical Insurance (URRBMI) scheme, which has been piloted in many counties and districts [21].

Given the relative backward and unbalanced economic and social development in China, the rural sector often cannot meet the need for chronic disease prevention and treatment, including VI. In rural China, the prevalence of VI is relatively high, while the access and use of eye care services are comparatively low [22]. Statistics show that in 2018, about 2.5 billion people in developing countries suffered from vision problems, with 30 % of them were Chinese. Especially in rural areas, poor access to timely services of vision correction and eye care increases the risk of VI. China has more than 28,000 ophthalmologists, which is five times the World Health Organization (WHO)’s recommendation of one ophthalmologist for every 250,000 people. Unfortunately, most specialized ophthalmologists are concentrated in cities and towns, leaving rural areas with a severe shortage of eye care resources [23].

With the development of urbanization and the changes in lifestyles, the Chinese government has gradually realized the importance of paying attention to the problem of visual impairment among rural adults in China [16, 24, 25]. Fortunately, the rapid development of health insurance systems led to programs, such as NCMS and URRBMI, that have been widely popularized and nearly covered all rural families since 2009. Implementing these health insurance policies effectively increased the availability of preventive, outpatient, and inpatient care for rural households [26–28]. With the help of NCMS, rural residents could get access to health service more conveniently, and their financial burden arising from seeking care could be alleviated, which greatly improves the equity in utilizing health services. The successful implementation of NCMS is inseparable from the crucial role played by the provider payment system [23, 29–33]. For eye diseases, NCMS has formulated specific clauses of insurance reimbursement, including the costs of medicine, examination, treatment, operation, bed, medical materials, blood transfusion, rehabilitation, etc., which provides financial guarantee for rural residents from the legal level. However, some studies found that out-of-pocket expenses and the associated financial burden remained unchanged or even increased after insurance enrollment [26–28, 34]. Health insurance enrollment’s impact on the overall physical health of rural population has been minimal [26]. Moreover, although the prevalence of NCMS increased significantly, the rate of eye care use in rural China was actually low, which is not conducive to improve the vision health of rural residents [13].

Since the studies globally on the effectiveness of health insurance in decreasing financial barriers to care and contributing to patients’ vision health have had mixed results, and few studies specifically focus on the impact of health insurance enrollment on vision health with the sample of China, especially the rural areas which generally have higher prevalence of VI, this paper thus tries to fill this gap in the literature. Consequently, based on the data from an epidemiological survey of vision health in rural China, which was conducted between June 2018 and October 2018, this study aims to: (1) examine whether health insurance enrollment impacts vision health outcomes in rural China; (2) analyze the impact of reimbursement rates of health insurance on vision health among the insured persons; (3) compare the heterogenous impacts of health insurance enrollment on vision health among people with different sex, age, education level, and whether with eye diseases; (4) examine whether health insurance enrollment moderate the impact of chronic physical conditions and basic eye conditions on vision health. Our findings will help expand the literatures on the effect of health insurance enrollment on health outcomes in rural China, with implications for reforming rural health insurance policies in China and other developing countries.

## Methods

### Study population

This cross-sectional study was based on the China Rural Glaucoma Prevention and Treatment Project, which was sponsored by China Disabled Persons Welfare Foundation and jointly organized and conducted in rural China by Tianjin Medical University from June 2018 to October 2018. The survey used a multistage stratified cluster sampling procedure to enroll a nationally representative sample population, with the targeted survey population aged six or older. In stage one, stratified by geographical region, ten provinces, autonomous regions, and municipalities were selected (including Shandong, Jiangsu, Ningxia, Shaanxi, Sichuan, Chongqing, Shanxi, Heilongjiang, Liaoning and Henan); in stage two, a large city and a mid-sized city were selected in random from each province or autonomous region; in stage three, two rural townships were randomly selected from each city; in stage four, one rural village community was randomly selected from each rural township; in stage five, individuals aged 6 years or older in each village were invited to participate in the baseline survey if they met the criterion of living in their current residence for one year or longer. A total of 52,041 participants from 40 villages were contacted, of which 48,398 completed the baseline survey questionnaire and eye examination (response rate: 93.0 %). The current study included only the adult respondents (age 18 or older). Moreover, some samples have missing values in some key variables. If we ignore the impact of missing values and directly conduct regression analysis, the regression results are likely to be biased. Therefore, we dropped the samples with missing values in the key variables (i.e., VI, health insurance enrollment, and reimbursement ratio), with a resulting study sample 28,787.

### Survey design

Standardized questionnaires were administered by trained interviewers to collect information on the respondents’ demographic status, socioeconomic conditions, health status, health-related behaviors, and health insurance enrollment. To ensure the accuracy and effectiveness of the survey, all interviewers received professional training. A pilot survey was conducted in Beizhen to ensure the appropriateness and feasibility of the questionnaire. The questionnaire is provided (see Additional file 1).

To collect eye examination data, the senior ophthalmologists conducted a series of ophthalmological examinations. Corrected visual acuity (CVA) was identified with a standard logarithmic visual acuity chart and recorded with decimals. Slit-lamp biomicroscopy and direct ophthalmoscopy were performed through dilated pupils, and an intraocular pressure check was conducted through non-contact tonometer.

### Variables

Table 1 shows the main variable definitions. To measure vision health, we referred to the VI definition proposed by the WHO. We scored VI from 0 to 5 according to the CVA in the better eye (BCVA), with higher values indicating poorer vision health.

**Table. 1:** Definitions and sample summary statistics of key variables

	Definitions	Full sample	Insured	Uninsured	P-value
VI	Scored according to BCVA ^a^, ranging from 0 to 5 ^b^	0.219	0.207	0.802	< 0.001
		(0.730)	(0.683)	(1.820)	
Insurance	Currently enroll in health insurance scheme (NCMS or URRBMI) (1 = Yes)	0.979	1.000	0.000	< 0.001
		(0.142)	(0.000)	(0.000)	
Reimbursement	Reimbursement ratio of health insurance (%) ^c^	-	0.829	-	-
		-	(0.079)	-	
**Chronical physical conditions**					
Hypertension	Had hypertension (1 = Yes)	0.234	0.235	0.192	0.013
		(0.423)	(0.424)	(0.394)	
Cardiopathy	Had cardiopathy (1 = Yes)	0.082	0.082	0.072	0.394
		(0.274)	(0.274)	(0.259)	
CI	Had cerebral infarction (1 = Yes)	0.048	0.048	0.055	0.417
		(0.215)	(0.214)	(0.229)	
Diabetes	Had diabetes (1 = Yes)	0.078	0.078	0.076	0.836
		(0.268)	(0.268)	(0.265)	
Hyperlipidemia	Had hyperlipidemia (1 = Yes)	0.046	0.047	0.034	0.136
		(0.210)	(0.211)	(0.180)	
Mental	Had mental illness such as anxiety or depression (1 = Yes)	0.006	0.006	0.032	< 0.001
		(0.079)	(0.076)	(0.176)	
**Basic eye conditions**					
Glaucoma	Had glaucoma (1 = Yes)	0.013	0.012	0.077	< 0.001
		(0.114)	(0.108)	(0.267)	
DR	Had diabetic retinopathy (1 = Yes)	0.009	0.009	0.030	< 0.001
		(0.095)	(0.093)	(0.171)	
MD	Had macular degeneration (1 = Yes)	0.007	0.006	0.037	< 0.001
		(0.083)	(0.079)	(0.189)	
RVO	Had retinal vein obstruction (1 = Yes)	0.005	0.004	0.030	< 0.001
		(0.069)	(0.065)	(0.171)	
Male	Gender (1 = Male)	0.412	0.410	0.477	0.001
		(0.492)	(0.492)	(0.500)	
Age	Age (year) = 2018 - year of birth	59.44	59.51	56.23	< 0.001
		(14.23)	(14.16)	(16.95)	
Single	Never married (1 = Yes)	0.042	0.040	0.133	< 0.001
		(0.200)	(0.195)	(0.340)	
Han	Ethnicity is Han (1 = Yes)	0.952	0.952	0.976	0.005
		(0.213)	(0.214)	(0.152)	
Child_Num	Number of children the respondent had	1.889	1.892	1.741	0.004
		(1.260)	(1.257)	(1.382)	
BMI	BMI (kg/m^2^) = Weight / (Height) ^2	23.24	23.24	23.36	0.403
		(3.421)	(3.422)	(3.358)	
Income	Personal annual income reported in six levels (≤ 10,000 yuan, 10,000–30,000 yuan, 30,000–50,000 yuan, 50,000–80,000 yuan, 80,000-120,000 yuan, ≥ 120,000 yuan), ranging from 1 to 6 ^d^	1.393	1.393	1.393	0.991
		(0.920)	(0.919)	(0.990)	
Education	Years of schooling according to six education level (no education, primary, junior, senior, undergraduate and postgraduate), ranging from 0 to 18 ^e^	6.421	6.404	7.224	< 0.001
		(4.663)	(4.661)	(4.691)	
Observation	Sample size	28,787	28,192	595	

Note: Data are from the epidemiological survey of glaucoma conducted from June 2018 to October 2018 in rural China. The reported statistics are the sample mean with standard deviation in parentheses. Column 3 shows the statistics of full sample, which covers all the respondents aged 18 and above. Column 4 to 5 correspond to the subsamples with health insurance (Insurance = 1) and without health insurance (Insurance = 0). Column 6 shows the results of Pearson’s chi-squared test, which studies whether the difference between the insured and the uninsured subsamples in each variable is statistically significant

Abbreviations: *BCVA* better corrected visual acuity; *BMI* body mass index; *CVA* corrected visual acuity; *MVI* moderate visual impairment; *NCMS* New Cooperative Medical Scheme; *NTB* near total blindness; *NVI* non-visual impairment; *PVI* profound visual impairment; *SVI* severe visual impairment; *TB* total blindness; *URRBMI* Urban and Rural Resident Basic Medical Insurance

^a^ Compared the CVA of both eyes, and recorded the CVA of the better eye as the BCVA. BCVA were recorded with decimals. Since the degree of VI is generally believed to be high if BCVA is less than 0.02, if BCVA is measured from finger counting, hand motion recognizing, light perception or no light perception, we value it as 0.01 for the record

^b^ If BCVA ≥ 0.3, 0 = NVI; if 0.1 ≤ BCVA < 0.3, 1 = MVI; if 0.05 ≤ BCVA < 0.1, 2 = SVI; if 0.02 ≤ BCVA < 0.05, 3 = PVI; if BCVA < 0.02, count fingers, hand motion, or light perception, 4 = NTB; if no light perception, 5 = TB

^c^ Calculated based on the reported inpatient reimbursement rate in the tier-1 designated medical institutions (primary care hospitals) of each county. Since the uninsured persons are not covered by health insurance, the uninsured subsample has no reimbursement rate. Only the reimbursement rate of the insured subsample has a value

^d^ 1 = Up to 10,000 yuan, 2 = 10,000–30,000 yuan, 3 = 30,000–50,000 yuan, 4 = 50,000–80,000 yuan, 5 = 80,000–120,000 yuan, 6 = More than 120,000 yuan

^e^ 0 = No education, 6 = Primary, 9 = Junior, 12 = Senior, 16 = Undergraduate, 18 = Postgraduate

Two key explanatory variables were used to measure health insurance. The first was whether the respondent was currently enrolled in any health insurance scheme. Since health insurance participation status may suffer from an endogeneity problem caused by self-selection for insurance enrollment, we used the instrumental variables (IV) method. Through extensively reviewing the literatures and considering the availability of data, we decided to extend the IV used in existing literatures, which is the county’s health insurance participation rate [35, 36], and use three county-level variables (i.e., the respondent’s county’s health insurance participation rate, per capita funding of health insurance, and duration of health insurance implementation) as the IVs for health insurance enrollment. The purpose of the IVs is to abstract from the unobservable characteristics of individuals that may be correlated with both health insurance enrollment and dependent variables. The rationality of using these three county-level variables as IV lies in the following two reasons. First, an individual’s insurance decision is strongly affected by the availability and generosity of the health insurance policies in the local area, thus there exists strong correlation between the three county-level variables and the individual participation in health insurance. Second, the availability and generosity of the county-level insurance schemes are determined ex-ante and exogenous to individuals’ insurance decisions, thus they are not likely to be correlated with the unobservable characteristics of individuals (i.e., random error term of the regression model). To ensure the rationality and effectiveness of the IVs, several IV tests were conducted before the regression analysis.

To measure health insurance’s impact on vision health in insured participants, health insurance reimbursement ratio was used as the second key independent variable. This further study is an extension of the previous question, which can reflets a richer policy significance. The reimbursement ratio is a nominal ratio, and is based on the reported inpatient reimbursement rate in tier-1 designated medical institutions (primary care hospitals) of each county, which is more accurately tracked and frequently used by rural residents. Different from the actual ex-post reimbursement ratio, the nominal reimbursement ratio is ex-ante ratio, which can better reflect the impact of the nominal reimbursement ratio stipulated by the ex-ante policy on vision health, from the perspective of policy formulation, and contribute to reform the relevant health insurance policies.

To examine whether health insurance enrollment moderates the impact of chronic physical conditions and basic eye conditions on vision health, we considered six chronic physical conditions of hypertension, cardiopathy, cerebral infarction (CI), diabetes, hyperlipidemia and mental illness, as well as four basic eye conditions of glaucoma, diabetic retinopathy (DR), macular degeneration (MD) and retinal vein obstruction (RVO).

The regression models also included a series of control variables, including sex, age, marital status, ethnicity, number of children, body mass index (BMI), income level, education level, and province.

### Statistical analysis

Given the discrete and sequential nature of the outcome variables that measure vision health (VI), we used ordered probit regression model. To address the potential endogeneity of the participation in health insurance, we used the IV method, with the three county-level variables as IVs. In estimating the impact of reimbursement ratio, since vision health in people who are not enrolled in health insurance is not affected by reimbursement ratio, we used Heckman selection model to address sample selection bias, which characterized the systematic differences between the health insurance participants and non-participants through a two-stage process. We also performed subsample regressions by sex (male vs. female), age (18–45 years, 46–60 years, 61–80 years, above 80 years), education level (primary school and below, junior high school, senior high school, college and above), and whether with basic eye diseases or not to explore the heterogeneity in our results. Robustness tests which control for chronic physical conditions and basic eye conditions were also conducted for the above regressions (see Additional file 1). In addition, we further investigated the moderating effect of health insurance by introducing interaction terms into the regression equations. Specifically, each variable of chronic physical conditions and basic eye conditions was firstly taken as the core explanatory variable to study its impact on vision health. Then, the moderator (health insurance enrollment), and the interaction term between the moderator and the corresponding chronic or basic eye condition were introduced into the first regression. Considering the endogeneity of health insurance enrollment, the IV method is used again. The benchmark models are constructed as follows:

1 $$\begin{array}{*{20}l} {VI}_{i}={\psi }_{0}+{\psi }_{1}{M}_{i}+\delta {Z}_{i}^{\prime}+{\epsilon}_{i} \end{array} $$

2 $$\begin{array}{*{20}l} {VI}_{i}={\rho }_{0}+{\rho }_{1}{M}_{i}+{\rho }_{2}{Insurance\_hat}_{i}+{\rho }_{3}{Insurance\_hat}_{i}*{M}_{i}+\tau {Z}_{i}^{\prime}+{\xi }_{i} \end{array} $$

where *M*_*i*_ denotes the variable of a certain chronic physical disease or basic eye disease. *Z*_*i*_ denotes the set of control variables. *Insurance_hat*_*i*_ denotes the predicted health insurance enrollment obtained by using the coefficients estimated from the first step of the IV method. *ξ*_*i*_ and *ε*_*i*_ denote the random error terms. *ρ*_*0*_ and *ψ*_*0*_ denote constant terms. *ψ*_*1*_, *ρ*_*1*_, *ρ*_*2*_, *ρ*_*3*_, *δ and τ* denote coefficients.

Through observing the sign and significance of the coefficients of the interaction term, we can judge whether health insurance enrollment plays a moderating role in the impact of chronic physical conditions and basic eye conditions on vision health. A detailed description of the statistical methods used in this study is provided (see Additional file 1).

## Results

### Description results of key variables

Table 1 shows the key variable summary statistics. Of the 28,787 respondents, most (28,192 respondents, or 97.9 %) participated in health insurance, and the average reimbursement rate was 82.9 %. The majority of respondents were female (58.8 %). The average age was 59.4 years, ranging from 18 to 104. Average BMI was in the normal category by the WHO standard (23.2 kg/m^2^). Most respondents were low income, with the average personal annual income under 30,000 yuan. 59 % (17,047 respondents) had a primary education or below. The average VI was 0.219. Among the chronic physical conditions, hypertension was the most common (23.4 %). Glaucoma was the most common basic eye condition (376 people, or 1.3 %).

### Comparison between the insured and uninsured groups

A comparison between the insured and uninsured groups revealed differences in key measures. Insured respondents had lower VI (0.207) than the uninsured respondents (0.802), suggesting that insured respondents had better vision than uninsured respondents. Women comprised 59 % of the insured group compared to 52.3 % in the uninsured group, indicating that women were more likely than men to participate in health insurance. Insured respondents were less educated (with 6.4 average years of schooling in the insured group and 7.2 in the uninsured group) and three years older on average (insured group average age was 59.5 compared to 56.2 in the uninsured group). Some chronic physical conditions (hypertension, cardiopathy, diabetes, and hyperlipidemia) were more commonly found in insured individuals than uninsured individuals, which may be a result of adverse selection in insurance enrollment behavior. Individuals with these conditions are generally more motivated to enroll in health insurance, aiming to better cope with the long-term financial stress of frequent medical needs. Insured respondents were less likely to have basic eye condition (glaucoma, DR, MD, and RVO), for the possible reasons that the higher cure rate of these eye conditions and the less obvious adverse selection caused by the shorter-term medical expenses in the insured respondents.

To determine whether the differences in the above characteristics between the two groups are statistically significant, Pearson’s chi-squared test was further conducted. As presented in Column 6 of Table 1, significant differences were found in VI, age, marital status, education level, mental illness, and four basic eye conditions (glaucoma, DR, MD, and RVO) of insured and uninsured respondents. However, the differences in sex, ethnicity, number of children, BMI, income level, and several chronic physical conditions (hypertension, cardiopathy, CI, diabetes, and hyperlipidemia) of insured and uninsured respondents were not significant.

### Health insurance and vision health

Table 2 shows the impact of health insurance on vision health. The probability of VI was 1.12 % lower for people participating in health insurance, which means that health insurance enrollment was associated with better vision health. After considering the endogeneity of health insurance participation, participating in health insurance reduced the probability of VI by 2.15 %, suggesting adverse selection in health insurance participation, where people with more severe VI are more likely to participate in health insurance. The reimbursement rate increasing by 1 % point may reduce the probability of worsening VI by 6.12 %, indicating that increasing the reimbursement rates may motivate people’s medical care use, thereby improving vision health in rural China’s insurance enrollees.

**Table. 2:** Effects of health insurance participation and reimbursement ratio on vision health

	Visual impairment		
	**Ordered probit regression**	**IV regression**	**Heckman selection model**
Insurance	-0.0112***	-	-
	(0.0012)	-	-
Insurance_hat ^a^	-	-0.0215***	-
	-	(0.0050)	-
Reimbursement	-	-	-0.0612***
	-	-	(0.0099)
Male	-0.0015***	-0.0016***	-0.0015***
	(0.0003)	(0.0003)	(0.0003)
Age	-0.0005***	-0.0004***	-0.0006***
	(0.0001)	(0.0001)	(0.0001)
Age^2	7.58e-06***	6.48e-06***	8.57e-06***
	(7.28e-07)	(7.07e-07)	(8.23e-07)
Han	-0.0036***	-0.0101***	-0.0030***
	(0.0008)	(0.0008)	(0.0009)
Single	0.0049***	0.0052***	0.0067***
	(0.0009)	(0.0009)	(0.0011)
Child_Num	0.0001	0.0005***	0.0001
	(0.0001)	(0.0001)	(0.0001)
Income	-0.0007	-0.0017***	-0.0008
	(0.0005)	(0.0005)	(0.0005)
BMI	-0.0013***	-0.0012***	-0.0012***
	(0.0002)	(0.0002)	(0.0002)
BMI^2	0.0000***	0.0000***	0.0000***
	(4.27e-06)	(4.31e-06)	(4.28e-06)
Education	-0.0004***	-0.0003***	-0.0004***
	(0.0000)	(0.0000)	(0.0000)
Province	Controlled	Controlled	Controlled
Observations	28,787	28,787	28,192
R-squared	0.1011	0.0769	0.0970

Note: Average marginal effect of all the explanatory variables are given, with robust standard errors shown in parentheses. ***, ** and * denote statistical significance at 1 %, 5 and 10 % levels, respectively

Abbreviations: *BMI* body mass index; *IV* instrumental variables

^a^ Insurance_hat represents the predicted health insurance enrollment obtained by using the coefficients estimated from the first step of the IV method

Of course, the premise of using the IV method is to ensure that the variable of health insurance participation is endogenous and that the selected IVs are effective and reasonable. Therefore, several necessary IV tests were conducted, including overidentification test, F-value test, underidentification test, weak identification test, IV redundancy test, IV heteroscedasticity test, and endogeneity test. The results of IV tests in Table 3 show that the variable of health insurance enrollment is indeed endogenous, which suggests the necessity of using IV method for regression analysis. Moreover, the selected IVs have passed the above IV tests, thus the problems of overidentification, underidentification, weak IV, and redundancy IV do not exist. In other words, these IVs satisfy exogeneity and are highly correlated with the endogenous variable of health insurance enrollment, thus they can be used as effective IVs in the subsequent regression analysis. A detailed explanation for the results of IV tests is provided (see Additional file 1).

**Table. 3:** Tests of instrumental variables

	Value of statistic	P-value
**1. Overidentification test of all instruments**		
Sargan statistic	0.0000	0.9999
**2. F-value test**		
F statistic	64.0900	0.0000
**3. Underidentification test**		
Kleibergen-Paap rk LM statistic	188.1150	0.0000
**4. Weak identification test**		
Cragg-Donald Wald F statistic	96.7100	
Kleibergen-Paap Wald rk F statistic	64.0900	
Stock-Yogo weak ID test critical values for K1 = 1 and L1 = 3:		
10 % maximal IV size	22.3000	
15 % maximal IV size	12.8300	
20 % maximal IV size	9.5400	
25 % maximal IV size	7.8000	
**5. IV redundancy test**		
IV tested: Inscover	25.4870	0.0000
IV tested: Insfund	168.3660	0.0000
IV tested: Insyear	122.8600	0.0000
**6. IV heteroscedasticity test**		
Pagan-Hall general test statistic	743.0980	0.0000
**7. Endogeneity test**		
Wu-Hausman F test	6.4473	0.0111
Durbin-Wu-Hausman chi-sq test	6.4488	0.0111

Note: Column 2 and 3 show the values and p-values of the statistics of each test respectively

Abbreviations: *IV* instrumental variables; *Inscover* the respondent’s county’s health insurance participation rate; *Insfund* the county’s per capita funding of health insurance; *Insyear* the county’s duration of health insurance implementation

### Heterogeneity analysis

Table 4 displays the between-group regression results. Men (-0.0235, *P* = 0.0002) benefit more from insurance enrollment than women (-0.0201, *P* = 0.0082) with respect to vision health. From the young adult group to the oldest group, the marginal effect of health insurance increased from − 0.0068 (*P* = 0.0394) to -0.0753 (*P* < 0.0001), indicating that the effect of health insurance enrollment on vision health is greater in older adults. The marginal effect on VI was most significant in people with lower education levels and weakened with increased education levels. People with basic eye diseases (-0.0503, *P* = 0.0022) benefit more from participating insurance than the people without basic eye diseases (-0.0262, *P* < 0.0001) with respect to vision health.

**Table. 4:** Heterogeneity in health insurance participation’s impact on vision health among people of different sex, age, education level, and basic eye condition

	Visual impairment					
	**Male**	**Female**	**18–45 years**	**46–60 years**	**61–80 years**	**Above 80 years**
Insurance_hat ^a^	-0.0235***	-0.0201***	-0.0068**	-0.0370***	-0.0380***	-0.0753**
	(0.0062)	(0.0076)	(0.0033)	(0.0087)	(0.0094)	(0.0310)
Observations	11,849	16,938	4,827	8,466	14,284	1,210
	**Primary school and below**	**Junior high school**	**Senior high school**	**College and above**	**With eye diseases**^**b**^	**Without eye diseases**
Insurance_hat	-0.0610***	-0.0561***	-0.0165**	0.0022	-0.0503***	-0.0262***
	(0.0095)	(0.0131)	(0.0066)	(0.0051)	(0.0164)	(0.0051)
Observations	17,047	7,082	2,702	1,956	746	28,041

Note: Average marginal effect of all the explanatory variables are given, with robust standard errors shown in parentheses. ***, ** and * denote statistical significance at 1 %, 5 and 10 % levels, respectively. Due to limited space, the results of control variables are not presented here

^a^ Insurance_hat represents the predicted health insurance enrollment obtained by using the coefficients estimated from the first step of the IV method

^b^ The eye diseases here refer to the eye diseases studied in the basic eye conditions in this paper, including glaucoma, diabetic retinopathy, macular degeneration, and retinal vein obstruction

### Moderating effect of health insurance

Columns 2 and 3 in Table 5 illustrate the impact of several chronic physical conditions and basic eye conditions on vision health. As for chronic physical conditions, the probability of VI was significantly higher for people with hypertension (0.0007, *P* = 0.0196), cardiopathy (0.0021, *P* < 0.0001), CI (0.0020, *P* = 0.0009), diabetes (0.0039, *P* < 0.0001), and mental illness (0.0114, *P* < 0.0001), which means that these chronic physical conditions were associated with worse vision health. However, the impact of developing hyperlipidemia on VI is insignificant (*P* = 0.3173). As for basic eye conditions, the probability of VI was significantly higher for people with glaucoma (0.0190, *P* < 0.0001), DR (0.0221, *P* < 0.0001), MD (0.0229, *P* < 0.0001), and RVO (0.0249, *P* < 0.0001), which means that all these basic eye conditions were associated with worse vision health.

**Table. 5:** Moderating effects of health insurance participation on the impacts of several chronic physical conditions and basic eye conditions on vision health

	Visual impairment			
	**Step 1**		**Step 2**	
**Chronic physical conditions**				
Hypertension	0.0007**	(0.0003)	0.0200	(0.0138)
Insurance_hat ^a^			0.0422***	(0.0155)
Hypertension * Insurance_hat			-0.0196	(0.0140)
Cardiopathy	0.0021***	(0.0005)	0.0295	(0.0217)
Insurance_hat			0.0405***	(0.0153)
Cardiopathy * Insurance_hat			-0.0279	(0.0220)
Cerebral infarction	0.0020***	(0.0006)	0.1224***	(0.0275)
Insurance_hat			0.0418***	(0.0155)
Cerebral infarction * Insurance_hat			-0.1225***	(0.0280)
Diabetes	0.0039***	(0.0005)	0.0429**	(0.0175)
Insurance_hat			0.0414***	(0.0154)
Diabetes * Insurance_hat			-0.0398**	(0.0177)
Hyperlipidemia	0.0007	(0.0007)	0.1355**	(0.0611)
Insurance_hat			0.0393**	(0.0153)
Hyperlipidemia * Insurance_hat			-0.1364**	(0.0617)
Mental	0.0114***	(0.0020)	0.1939***	(0.0561)
Insurance_hat			0.0420***	(0.0159)
Mental * Insurance_hat			-0.1873***	(0.0569)
**Basic eye conditions**				
Glaucoma	0.0190***	(0.0015)	0.1531***	(0.0503)
Insurance_hat			0.0434***	(0.0164)
Glaucoma * Insurance_hat			-0.1369***	(0.0510)
Diabetic retinopathy	0.0221***	(0.0015)	0.1754***	(0.0541)
Insurance_hat			0.0351**	(0.0144)
Diabetic retinopathy * Insurance_hat			-0.1560***	(0.0546)
Macular degeneration	0.0229***	(0.0017)	0.0583	(0.0401)
Insurance_hat			0.0392**	(0.0152)
Macular degeneration * Insurance_hat			-0.0362	(0.0406)
Retinal vein obstruction	0.0249***	(0.0021)	0.2230***	(0.0825)
Insurance_hat			0.0393**	(0.0154)
Retinal vein obstruction * Insurance_hat			-0.2018**	(0.0834)

Note: Average marginal effect of all the explanatory variables are given, with robust standard errors shown in parentheses. ***, ** and * denote statistical significance at 1 %, 5 and 10 % levels, respectively. Due to limited space, the results of control variables are not presented here

^a^ Insurance_hat represents the predicted health insurance enrollment obtained by using the coefficients estimated from the first step of the IV method

Results from analyses of the moderating effect of health insurance enrollment on the impact of several chronic physical conditions and basic eye conditions on vision health are presented in Columns 4 and 5 of Table 5. The coefficients of the interaction terms between health insurance enrollment and CI (-0.1225, *P* < 0.0001), diabetes (-0.0398, *P* = 0.0245), hyperlipidemia (-0.1364, *P* = 0.0271), mental illness (-0.1873, *P* = 0.0010), glaucoma (-0.1369, *P* = 0.0073), DR (-0.1560, *P* = 0.0043), and RVO (-0.2018, *P* = 0.0155) were significantly negative, while the coefficients of the interaction terms between health insurance enrollment and hypertension (*P* = 0.1615), cardiopathy (P = 0.2047), and MD (*P* = 0.3726) were insignificant. Moreover, the coefficients of health insurance enrollment were significantly positive in these regressions, which is different from the negative coefficients presented in Table 2 (-0.0215, *P* < 0.0001). Combined with the results in Table 2, it can be seen that the positive effect of participating health insurance on visual health obtained in Table 2 is not directly, but is indirectly realized through moderating and reducing the adverse impacts of some chronic physical and eye diseases on visual health. The adverse selection problem of rural residents was also verified again by the positive coefficients of health insurance enrollment.

## Discussion

Based on a nation-wide epidemiological survey of vision health conducted in rural China in 2018, this study explored the impact of health insurance enrollment and reimbursement ratio on vision health, based on which, further examined the moderating role played by health insurance enrollment in the impact of several chronic physical and basic eye conditions on vision health. We found that participating in health insurance and a higher reimbursement rate were positively correlated with better vision health. Moreover, through comparing the two regression results of the used and unused instrumental variables method, the adverse selection problem of Chinese rural residents in deciding whether to participate in health insurance was confirmed. Our findings reinforce past studies demonstrating that participating in health insurance is associated with lower VI [14–18]. However, a study based on a US sample found that the frequency of eye care utilization by people with health insurance was positively associated with VI severity, which reflects the adverse selection of insurance participants [19]. Adverse selection means that people with a higher demand for medical services are more likely to enroll in health insurance, especially among the poor rural residents [12].

Through the heterogeneity analysis, the current study indicated that health insurance enrollment generally had a more significant impact on promoting vision health in vulnerable groups, such as middle-age and older adults, people with low education levels, and people with eye diseases. An existing study also based on rural Chinese adults which was conducted in Handan found that low vision was related to less education and older age [37], and a similar conclusion have also been demonstrated in another study conducted in Shaanxi, China [38]. Actually, not only the older people and less educated people, but also the female was also found to be more likely to have VI [22, 39, 40]. Since the pervasive inequality exists in the vulnerable populations, more care should be given to the vulnerable groups to a greater extent in the process of formulating health insurance, so as to reduce the inequality [24]. The greater impact of health insurance on promoting vision health in vulnerable groups demonstrates its effectiveness in improving the equity in utilization of health services. Besides, through comparing the two subsamples of with and without basic eye diseases, the remedial care impact of health insurance on vision health could be separated. This study found that the impact of health insurance enrollment on vision health is greater among people with basic eye diseases, which means that through participating in health insurance, people with basic eye diseases can get generally more medical assistance and health service, as well as more thoughtful remedial care, which can improve the vision health of these people more significantly.

We also found that the chronic physical diseases, including hypertension, cardiopathy, CI, diabetes, and mental illness, have significant negative impacts on the visual health in rural China. China was found to lag behind more developed countries in controlling chronic physical diseases, including hypertension and diabetes, even after 10 years of healthcare reform [41]. Existing studies have demonstrated the effects of chronic physical conditions on vision health. The burden of some chronic physical conditions on people’s lives is longstanding, including overweight, hypertension, diabetes, etc., which pose a threat to vision health [42]. High blood pressure can cause substantial vision damage [43, 44]. VI is also commonly found in patients with diabetes [18, 37, 45]. Vision problems often coexist with hypertension and dyslipidemia, which may increase the risk of CI; moreover, people with longer histories of diabetes and hypertension usually have a higher level of VI [46]. Cardiovascular diseases’ impact on vision health is difficult to gauge because of the confounding effect of underlying conditions such as hypertension and dyslipidemia, but controlling blood pressure often helps improve overall vision [47]. VI is also found to be associated with mental illness [48].

In addition, the intuitive conclusion that basic eye diseases (i.e., glaucoma, DR, MD, and RVO) generally increase the risk of VI was also confirmed in this study. Many previous studies have shown the significant impact of basic eye conditions on VI outcomes. A US-based study found that the most common eye conditions that cause VI include MD, DR, glaucoma, cataract, and other retinal disorders [49, 50]. Glaucoma, cataract, and DR were also leading causes of blindness, based on the National Eye Survey of Trinidad and Tobago [18]. DR and glaucoma were two common causes of bilateral visual impairment, based on a study of rural areas in Tianjin, China [22]. The effects of DR, MD, and glaucoma on VI are especially strong [51–56]. Retinal diseases, such as DR and RVO, are one of the primary causes of VI and blindness in cataract-operated eyes [57]. MD has a serious adverse impact on daily life as it gradually damages central vision [58]. Glaucoma is usually difficult to detect [59], but it can gradually destroy nerve heads and other visual sensory structures [60, 61]. Without timely and proper treatment, it is likely to cause incurable permanent VI [11, 62]. These findings present strong evidence that the occurrence of basic eye conditions, such as glaucoma, DR, MD, and RVO is detrimental to people’s vision health.

The moderating effect of health insurance enrollment was also verified in this study. Specifically, the adverse effects of several chronic diseases and eye diseases on visual health were significantly reduced through participating in health insurance, including CI, diabetes, hyperlipidemia, mental illness, glaucoma, DR, and RVO. First, through participating in health insurance, rural residents can get access to more timely and professional medical services, so that patients can receive more effective treatment [23]. Second, health insurance can greatly reduce the financial pressure of rural residents, so that they will not delay treatment due to economic pressure [12, 29]. Moreover, some studies have shown that chronic diseases, such as CI, diabetes, hyperlipidemia, mental illness, are difficult to be cured and often generate long-term medical costs, which makes the moderating effect of health insurance enrollment more obvious in the negative effects of these diseases [16]. Third, health insurance participants usually have a stronger sense of health care and are more active in accepting regular physical examinations, which makes some chronic diseases and eye diseases early detected and treated [13].

In addition, after the introducing of interaction terms in the regression, the coefficients of health insurance enrollment were significantly positive, which is obviously different from the negative coefficient regression results without the introduction of interaction terms (Table 2). However, this doesn’t mean that these results are contradictory. The negative relationship between health insurance enrollment and VI in Table 2 is due to the fact that the direct impacts of chronic physical and basic eye diseases on VI were not taken into account in the regression of Table 2, thus exaggerating the direct impact of health insurance enrollment on VI to some extent. Through introducing the interaction term, the direct impact of chronic physical and basic eye diseases on VI, the direct impact of health insurance enrollment on VI, and the moderating effect of health insurance enrollment were separated, which not only proves that participating in health insurance can reduce the negative effects of some chronic physical and basic eye diseases on VI, but also indicates the existence of adverse selection in rural residents’ decision on whether to participate in health insurance. Specifically, people with more severe VI are more likely to enroll in health insurance. Since these people require more medical services such as surgery and regular check-ups, as well as medical expenses such as drugs and medical consumables, they are more inclined to let the government share their financial pressure by participating in health insurance, after fully weighing the costs and benefits [12]. In contrast, people without vision problems are insignificantly affected in daily lives and have less need for medical services, thus they have little incentive to enroll in health insurance.

Some limitations must be acknowledged. First, this study is based on a cross-sectional design of survey. However, analyzing cross-sectional data cannot precisely judge the causal relationship between variables. Therefore, further follow-up studies are necessary to conduct longitudinal study in the future. Second, in this current study, since some samples have missing values in key variables, a number of observations were dropped for regression analysis, which greatly reduced the sample size and increased the potential for estimation bias. Third, limited by the availability of data, we were unable to focus on the effects of health insurance on the outcomes of diseases, by channels such as increasing the utilization of health service, benefit coverage for eye disease, as well as economic protection. Thus, these need to be further examined in future studies. Fourth, this study only focuses on the impact of health insurance on visual health in rural China, but not in urban China and other countries. More comparative studies on rural and urban areas, as well as China and other countries could be carried out in the future.

## Conclusions

This study is the first to examine whether and how health insurance influences adult vision health outcomes in rural China. No prior studies have specifically addressed this issue, despite the importance of vision health for the rural population. Although previous studies have demonstrated that rural health insurance has no significant impact on the overall physical health of rural populations [26], we found that both participating in health insurance and increased reimbursement ratios contributed to improving vision health in China’s rural adults, based on a vision health epidemiological survey conducted in rural China during 2018. Health insurance has the most significant effect in men, middle-aged and older adults, people with low education levels, and people with basic eye diseases. Heath insurance enrollment moderates the impacts of several chronic physical conditions (i.e., CI, diabetes, hyperlipidemia, and mental illness) and basic eye conditions (i.e., glaucoma, DR, and RVO) on vision health.

These findings have several implications for continuing reform of China’s health insurance policies. First, the existing rural health insurance schemes should be further promoted and expanded, and more reasonable and effective reimbursement policies for eye disease diagnosis, treatment, and medication should be formulated in China’s large rural areas, because higher coverage rates and reimbursement rates are shown to contribute positively to vision health and certain other physical health conditions. Policymakers should be aware of the insurance-related moral hazard to reduce the potential welfare loss associated with policy reforms. This is especially true for China’s large and complex health insurance system, for which provider-induced moral hazards are a prevalent phenomenon [27]. Because of information asymmetry, health care providers, especially those who are paid on a fee-for-service basis, have incentives to encourage unnecessary tests and treatments, which results in considerable waste of medical resources and prevents health insurance from effectively serving its purpose of reducing patients’ out-of-pocket spending [34]. Therefore, health insurance expansion reforms should be accompanied by reimbursement payment reforms (e.g., payment for performance, capitation-based reimbursement, etc.) to further improve vision health in the population.

Second, special attention should be paid to vulnerable groups in the rural sector, when formulating health insurance policies. Extra financial and social support should be provided to the vulnerable populations, including older adults, the very poor, the less educated, and the chronically ill or disabled groups. Research shows that catastrophic medical insurance and medical aid can effectively supplement basic social health insurance schemes, reducing the average proportion of out-of-pocket expenses after reimbursement by about 10 % [21]. Since the 2009 launch of major health-care reform, the Chinese government has quadrupled its funding for health [63], aiming to achieve universal health insurance coverage for disadvantaged groups, with priorities for basic primary care and disease prevention. These policy goals, when achieved, may help to improve overall health and vision health in vulnerable groups. Constrained by lower income and lack of medical knowledge, the rural population in China (especially its most disadvantaged groups) do not have continuity in the diagnosis and treatment of eye diseases, which prevents patients from receiving follow-up medical advice, timely medication, and regular support. This increases the risk of vision impairment or blindness for those with the highest health risks. Therefore, a long-term management mechanism should be established, and regular screening and preventive practices for basic eye conditions should be available to vulnerable groups. Following treatment, regular follow-up visits and counseling should be available. Patient files and corresponding databases should be constructed to facilitate early detection, early diagnosis, and early treatment of eye diseases.

Third, capacity building efforts should be developed to strengthen China’s vision care system. The current level of primary medical services in China is relatively weak, especially in rural areas. Most medical services in these areas lack specialized ophthalmologists, and their diagnostic and treatment capabilities for many eye diseases are limited. Therefore, professional medical and health teams should be cultivated for rural areas; the ability of grass-roots medical staff to diagnose and treat eye diseases should be improved through receiving regular training. Given the backward transmission methods disseminating medical knowledge and information in rural China, local residents usually lack awareness of blindness prevention and eye care [63]. Therefore, large-scale health education campaigns should be launched to raise rural people’s awareness of common eye diseases, improve their knowledge of eye health, and help them form good eye-using habits.

## Supplementary Information

**Additional file 1:**

## Data Availability

The datasets used during the current study are available from the corresponding author on reasonable request.

## Abbreviations

BCVA: Better corrected visual acuity
BMI: Body mass index
CI: Cerebral infarction
CVA: Corrected visual acuity
DR: Diabetic retinopathy
IV: Instrumental variables
MD: Macular degeneration
MVI: Moderate visual impairment
NCMS: New Cooperative Medical Scheme
NTB: Near total blindness
NVI: Non-visual impairment
PVI: Profound visual impairment
RVO: Retinal vein obstruction
SVI: Severe visual impairment
TB: Total blindness
UEBMI: Urban Employee Basic Medical Insurance
URBMI: Urban Resident Basic Medical Insurance
URRBMI: Urban and Rural Resident Basic Medical Insurance
VI: Visual impairment
WHO: World Health Organization
