# Supplementary material for: Health insurance enrollment and vision health in rural China: an epidemiological survey
Source: BMC Health Serv Res. 2021 Jul 31;21:761. doi: 10.1186/s12913-021-06754-0 (PMC8325859; doi:10.1186/s12913-021-06754-0)
Supplement: Supplementary file 1 — Additional file 1: [file 12913_2021_6754_MOESM1_ESM.docx]

**Additional File 1**

**A1: Detailed description of the statistical methods**

Given the discrete and sequential nature of the outcome variables that measure vision impairment (VI), we used ordered regression models, which assume that the observed VI levels for individual *i* are determined by an unobserved continuous latent variable ${VI}_{i}^{*}$, such that if $\mu_{j-1}<{VI}^{*}<\mu_{j}$ then $VI=j\left( j=0,1,2,3,4,5 \right)$, where $\mu_{-1},\ldots,\mu_{5}$ denote the thresholds. Therefore, we can get the regression equation of the latent variable ${VI}_{i}^{*}$ as follows:

${VI}_{i}^{*}=\beta_{0}+\beta_{1}{Insurance}_{i}+\gamma X_{i}^{'}+\varepsilon_{i}$ (1)

where ${Insurance}_{i}$ denotes whether respondent *i* is currently enrolled in health insurance. $X_{i}$ denotes a set of control variables including gender, age, marital status, ethnicity, number of children, BMI, income level and education level. $\beta_{1}$ is the key parameters to be estimated and it represents the impact of insurance enrollment on vision health. $\varepsilon_{i}$ is the random error term, and is assumed to follow either a standard normal distribution (the Ordered Probit Regression) or a standard logistic distribution (the Ordered Logistic Regression). Therefore, the conditional probability density function of VI and the corresponding regression equation of latent variable are:

$Pr\left( VI=j\left| X \right. \right)=Pr\left( \mu_{j-1}<{VI}^{*}<\mu_{j}\left| X \right. \right)=\Phi\left[ \mu_{j}-f\left( X \right)\left| X \right. \right]-\Phi\left[ \mu_{j-1}-f\left( X \right)\left| X \right. \right]$ (2)

$f\left( X \right)=\beta_{0}+\beta_{1}{Insurance}_{i}+\gamma X_{i}^{'}$ (3)

where $\Phi\left( \cdot\right)$ denotes the cumulative distribution function of a standard normal distribution for the Ordered Probit Regression, which is the main model we use for our analysis; $\Phi\left( \cdot\right)$ denotes the cumulative distribution function of logistic distribution for the Ordered Logistic Regression, which we use for robustness check purpose. To address the potential endogeneity of the participation in health insurance, we use the instrumental variables (IV) method in combination with the Ordered Probit models, with the IVs being the three county-level variables (the health insurance participation rate of the respondent’s county, the per capita funding of health insurance of the county, and the length of time since the county started to implement health insurance policy). The IV estimation is carried out using the two-step control function (CF) approach in a quasi-MLE framework as proposed by Wooldridge (2014). Compared to the traditional two-step CF approach of Wooldridge (2010), this estimation procedure allows for more flexible assumptions on the functional forms and error distributions, and it provides more robust estimates of the average partial effects in non-linear models (such as the current Ordered Probit model) where endogeneity is of concern.

In estimating the impact of reimbursement ratio of health insurance, the problem of sample selection bias needs to be considered, as the vision health of people who are not enrolled in health insurance is not affected by the reimbursement ratio. Following the econometric literature, we use the Heckman Selection Model to address the above issue, which explicitly characterize the systematic differences between the participants and non-participants of health insurance through the following two-stage process. In stage one, the participation equation (Equation (4)) characterizes the decision of health insurance enrollment i by a Probit model:

${Insurance}_{i}=\alpha Z_{i}^{'}+\mu_{i}$ (4)

where $Z_{i}$ denotes the set of variables that influence the health insurance participation decision, and $\mu_{i}$ denotes the random error term that follows the standard normal distribution. To satisfy the exclusion restriction condition for the Heckman Selection Model, $Z_{i}$ includes the three county-level instrumental variables as well as all the variables in matrix $X_{i}$ of Equation (1). Based on the OLS estimation results of the first step, the Inverse Mills Ratio can be calculated as follows:

$\hat{\lambda_{i}}=\frac{\phi\left( \hat{\alpha}Z_{i}^{'}/\sigma\right)}{\Phi\left( \hat{\alpha}Z_{i}^{'}/\sigma\right)}$ (5)

where $\phi\left( \cdot\right)$ and $\Phi\left( \cdot\right)$ denote the probability density function and the cumulative distribution function of the standard normal distribution, respectively.

In the second stage of the Heckman Selection Model, the intensity equation (Equation (6)) that characterizes the impact of health insurance reimbursement rate on the vision health outcome would include $\hat{\lambda_{i}}$ as an additional control variable to address the sample selection bias. Therefore, the regression model in the second stage is constructed as follows:

$E\left( {VI}_{i}\left| {Insurance}_{i}=1 \right. \right)= \kappa{ratio}_{i}+\theta X_{i}^{'}+\omega\lambda_{i}+\upsilon_{i}$ (6)

where ${ratio}_{i}$ indicates the health insurance reimbursement rate, $\kappa$ denote the key parameters to be estimated, and $\nu_{i}$ denotes the random error term. A significance test of parameter $\omega$ would indicates whether the sample selection bias is significant and whether the Heckman selection model is an appropriate correction method.

In addition to the above benchmark models, we also performed subsample regressions according to different genders, ages, education levels, and whether with basic eye diseases to explore the heterogeneity in the impact of health insurance participation on vision health. For each subsample, with full consideration of the endogenous problem of health insurance participation, the IV method is used to examine the impact of health insurance on VI. Robustness tests which control for chronic physical conditions and basic eye conditions were also conducted for main regressions and subsample regressions.

In order to further investigate whether health insurance enrollment moderates the impact of chronic physical conditions and basic eye conditions on vision health, the interaction term was introduced into the regression equations. Specifically, each variable of chronic physical conditions and basic eye conditions was firstly taken as the core explanatory variable to study its impact on vision health (Equation (7)). Then, the moderator (health insurance enrollment), and the interaction term between the moderator and the corresponding chronic or basic eye condition were introduced into Equation (7) and generated the Equation (8). Considering the endogeneity of health insurance enrollment, the IV method is used again. The benchmark models are constructed as follows:

${VI}_{i}=\psi_{0}+\psi_{1}M_{i}+\delta Z_{i}^{'}+\epsilon_{i}$ (7)

${VI}_{i}=\rho_{0}+\rho_{1}M_{i}+\rho_{2}{Insurance\_hat}_{i}+\rho_{3}{Insurance\_hat}_{i}*M_{i}+\tau Z_{i}^{'}+\xi_{i}$ (8)

where *M_i_* denotes the variable of a certain chronic physical disease or basic eye disease. *Z_i_* denotes the set of control variables. *Insurance_hat_i_* denotes the predicted health insurance enrollment obtained by using the coefficients estimated from the first step of the IV method. *ξ_i_* and *ε_i_* denote the random error terms. *ρ_0_* and *ψ_0_* denote constant terms. *ψ_1_*, *ρ_1_*, *ρ_2_*, *ρ_3_*, *δ and τ* denote coefficients. Furthermore, the issue of endogeneity of the core explanatory variable (health insurance enrollment) still cannot be ignored here, so we also use the IV method to estimate.

Through observing the sign and significance of the coefficients of the interaction term, we can judge whether health insurance enrollment plays a moderating role in the impact of chronic physical conditions and basic eye conditions on vision health.

**A2: Detailed explanation for the results of** **instrumental variables tests**

1. The precondition of using the instrumental variable (IV) method is to ensure the effectiveness of the IVs. Effective IVs should be exogenous and highly correlated with the endogenous explanatory variable. Therefore, to ensure the effectiveness of IVs, we have conducted the following tests for the selected IVs in this paper.
2. The overidentification test is first conducted to examine whether all the IVs satisfied exogeneity, that is, were not correlated with disturbance items. The null hypothesis of this test is that all the IVs are exogenous. The results for Sargan statistic in Table 3 show that the p-value is 0.9999, and the null hypothesis is acceptable. Therefore, it can be concluded that these IVs satisfy the hypothesis of exogeneity and are independent of the disturbance items.
3. Next, the second condition of effective IVs is further investigated, that is, the high correlation between IVs and endogenous explanatory variable. In view of this, we have carried out the following tests:
4. First, we conducted a simple F-value test. By observing the F statistic obtained from the estimation results of the first-stage regression, it can be seen that the p-value is 0.0000, which means that the selected IVs are highly correlated with the endogenous explanatory variable.
5. To use the IV method, the rank condition must be satisfied. The meaning of rank condition is that IVs are correlated with the endogenous explanatory variable, which can be tested through underidentification test. The null hypothesis of this test is that the problem of underidentification exists, that is, the IVs are not correlated with endogenous explanatory variable. In general, if the assumption that the disturbance term is independent identically distributed (IID) is not made, that is, heteroscedasticity is allowed to exist, then Kleibergen-Paap rk LM statistic should be used for underidentification test (Kleibergen and Paap, 2006). The results for Kleibergen-Paap rk LM statistic in Table 3 show that the p-value is 0.0000, and the null hypothesis is rejected. Therefore, it can be concluded that these IVs satisfy the hypothesis of exogeneity and are independent of the disturbance items. This result means that the selected IVs do not have the problem of underidentification, the rank condition is satisfied, and the IVs are correlated with the endogenous explanatory variable.

However, even if there is no underidentification problem, the problem of weak IVs may exist. Specifically, weak correlation may exist between some IVs and the endogenous explanatory variable, which will make the estimated asymptotic variance too large, thus greatly reducing the accuracy of estimation by the IV method.

1. Therefore, we further conducted the weak identification test. The null hypothesis of this test is that IVs are strongly correlated with the endogenous explanatory variable. In general, if the assumption that the disturbance term is IID is not made, that is, heteroscedasticity is allowed to exist, then Kleibergen-Paap Wald rk F statistic should be used for weak identification test. Meanwhile, the minimum eigenvalue statistics provided by Stock and Yogo (2005) could be used as the thresholds in the weak identification test. The results for Kleibergen-Paap Wald rk F statistic in Table 3 show that if the significance of endogenous explanatory variable in the structural equation is tested with a nominal size significance of 5%, assuming that the acceptable true size of significance is no more than 10%, then the null hypothesis can be rejected. This is because the Kleibergen-Paap Wald rk F statistic (64.09) is greater than the corresponding threshold of the 10% true significance size (22.30). In addition, if the disturbance term is assumed IID, that is, no heteroscedasticity exists, then Cragg-Donald Wald F statistic should be used for weak identification test (Cragg and Donald, 1993), with the thresholds still come from Stock and Yogo (2005). The results for Cragg-Donald Wald F statistic in Table 3 is the same as the above results of Kleibergen-Paap Wald rk F statistic.
2. To further investigate the problem of weak IVs and test whether the selected IVs would not improve the asymptotic efficiency of the estimator, we also conducted redundancy tests. The null hypothesis of this test is that the examined IV is redundant. The three IVs have been conducted redundancy test successively. The results of redundancy test for all the three IVs in Table 3 show that the null hypothesis is rejected (p-value = 0.0000).
3. Another prerequisite for the use of IV method is the existence of endogenous explanatory variable. In general, we use Hausman specification test to test the endogeneity of the explanatory variable (Hausman, 1978). The traditional Hausman test is based on the assumption of homoscedasticity. However, the Hausman test cannot be used if the problem of heteroscedasticity exists. Therefore, we conducted the IV heteroscedasticity test (Pagan and Hall, 1983). The results for Pagan-Hall general test statistic in Table 3 show that the p-value is 0.0000, which rejects the null hypothesis that disturbance is homoscedastic, thus the problem of heteroscedasticity exists.

Due to the problem of heteroscedasticity, we conducted the endogeneity test which is robust to heteroscedasticity, known as the DWH test (Dubin-Wu-Hausman Test) (Durbin, 1954; Wu, 1974; Hausman, 1978). The null hypothesis of this test is that the variable tested is exogenous. The results of DWH test in Table 3 show that the null hypothesis is rejected (p-value = 0.0111), which means that with fully consideration of the heteroscedasticity, health insurance enrollment is still an endogenous explanatory variable, and heteroscedasticity has no significant impact on this conclusion.

**A3: Robustness tests** **with chronic physical conditions and basic eye conditions controlled for**

**Table A3-1: Effects of health insurance participation and reimbursement ratio on vision health (with chronic physical conditions and basic eye conditions controlled for)**

|  | **Visual impairment** | | |
| --- | --- | --- | --- |
|  | **Ordered probit regression** | **IV regression** | **Heckman selection model** |
| Insurance | -0.0085*** |  | - |
|  | (0.0010) |  | - |
| Insurance_hat ^a^ |  | -0.0280*** |  |
|  |  | (0.0047) |  |
| Reimbursement | - | - | -0.0561*** |
|  | - | - | (0.0095) |
| Male | -0.0011*** | -0.0014*** | -0.0011*** |
|  | (0.0003) | (0.0003) | (0.0003) |
| Age | -0.0006*** | -0.0005*** | -0.0007*** |
|  | (0.0001) | (0.0001) | (0.0001) |
| Age^2 | 8.02e-06*** | 6.80e-06*** | 8.42e-06*** |
|  | (7.30e-07) | (7.04e-07) | (8.00e-07) |
| Han | -0.0047*** | -0.0102*** | -0.0047*** |
|  | (0.0008) | (0.0008) | (0.0009) |
| Single | 0.0039*** | 0.0036*** | 0.0044*** |
|  | (0.0008) | (0.0009) | (0.0010) |
| Child_Num | 0.0001 | 0.0004*** | 0.0001 |
|  | (0.0001) | (0.0001) | (0.0001) |
| Income | -0.0006 | -0.0010** | -0.0007 |
|  | (0.0005) | (0.0004) | (0.0005) |
| BMI | -0.0012*** | -0.0012*** | -0.0011*** |
|  | (0.0002) | (0.0002) | (0.0002) |
| BMI^2 | 0.0000*** | 0.0000*** | 0.0000*** |
|  | (4.27e-06) | (4.32e-06) | (4.30e-06) |
| Education | -0.0004*** | -0.0003*** | -0.0004*** |
|  | (0.0000) | (0.0000) | (0.0000) |
| Hypertension | -0.0001 | 0.0000 | -0.0002 |
|  | (0.0004) | (0.0004) | (0.0004) |
| Cardiopathy | 0.0012** | 0.0002 | 0.0010** |
|  | (0.0005) | (0.0005) | (0.0005) |
| Cerebral infarction | 0.0008 | 0.0008 | 0.0008 |
|  | (0.0006) | (0.0006) | (0.0006) |
| Diabetes | 0.0027*** | 0.0028*** | 0.0028*** |
|  | (0.0005) | (0.0005) | (0.0005) |
| Hyperlipidemia | -0.001 | -0.0011 | -0.001 |
|  | (0.0007) | (0.0007) | (0.0007) |
| Mental | 0.0038** | 0.0025 | 0.0052** |
|  | (0.0018) | (0.0020) | (0.0021) |
| Glaucoma | 0.0132*** | 0.0115*** | 0.0143*** |
|  | (0.0013) | (0.0013) | (0.0018) |
| Diabetic retinopathy | 0.0136*** | 0.0137*** | 0.0135*** |
|  | (0.0012) | (0.0012) | (0.0012) |
| Macular degeneration | 0.0119*** | 0.0116*** | 0.0123*** |
|  | (0.0015) | (0.0015) | (0.0016) |
| Retinal vein obstruction | 0.0035* | 0.0035* | 0.0042** |
|  | (0.0018) | (0.0019) | (0.0021) |
| Province | Controlled | Controlled | Controlled |
| Observations | 28,787 | 28,786 | 28,787 |
| R-squared | 0.1309 | 0.1099 | 0.1290 |

Note: Average marginal effect of all the explanatory variables are given, with robust standard errors shown in parentheses. ***, ** and * denote statistical significance at 1%, 5% and 10% levels, respectively.

Abbreviations: BMI=body mass index. IV=instrumental variables.

^a^ Insurance_hat represents the predicted health insurance enrollment obtained by using the coefficients estimated from the first step of the IV method.

**Table A3-2: Heterogeneity in health insurance participation’s impact on vision health among people of different sex, age, education level, and basic eye condition (with chronic physical conditions and basic eye conditions controlled for)**

|  | **Visual impairment** | | | | | |
| --- | --- | --- | --- | --- | --- | --- |
|  | **Male** | **Female** | **18 – 45 years** | **46 – 60 years** | **61 – 80 years** | **Above 80 years** |
| Insurance_hat ^a^ | -0.0302*** | -0.0299*** | -0.0099** | -0.0222*** | -0.0274*** | -0.0803*** |
|  | (0.0065) | (0.0069) | (0.0039) | (0.0078) | (0.0083) | (0.0286) |
| Observations | 11,849 | 16,938 | 4,827 | 8,466 | 14,284 | 1,210 |
|  | **Primary** **school and below** | **Junior** **high school** | **Senior high school** | **College and above** | **With eye diseases ^b^** | **Without eye diseases** |
| Insurance_hat | -0.0623*** | -0.0532*** | -0.0221*** | -0.0020 | -0.0623*** | -0.0273*** |
|  | (0.0082) | (0.0119) | (0.0079) | (0.0032) | (0.0170) | (0.0051) |
| Observations | 17,047 | 7,082 | 2,702 | 1,956 | 746 | 28041 |

Note: Average marginal effect of all the explanatory variables are given, with robust standard errors shown in parentheses. ***, ** and * denote statistical significance at 1%, 5% and 10% levels, respectively. Due to limited space, the results of control variables are not presented here.

^a^ Insurance_hat represents the predicted health insurance enrollment obtained by using the coefficients estimated from the first step of the IV method.

^b^ The eye diseases here refer to the eye diseases studied in the basic eye conditions in this paper, including glaucoma, diabetic retinopathy, macular degeneration, and retinal vein obstruction.

**A4: Questionnaire for the project of prevention and treatment of glaucoma**

Code： Province： County： Town / Village：

ID card No.： Tel.:

**Part 1A. General Information (For Adults)**

| Name |  | | Gender | | | □ Male □ Female | | | Age |  |
| --- | --- | --- | --- | --- | --- | --- | --- | --- | --- | --- |
| Marital status | □ Unmarried □ Cohabitation  □ Married/Common-law □ Others  □ Divorced/Separated/Widowed | | | | | | | | Ethnicity | □ Han  □ Hui  □ Manchu  □ Others |
| Number of Children |  | Number of Family Members | | |  | | Medical Insurance | | □ Yes (NCMS, URRBMI)  □ No | |
| Place of Residence | Province City (County) | | | | □ Registered Rural Residents □ Registered Urban Residents | | | | | |
| Occupation | □ Student □ Worker □ Farmer □ Individual Business □ Public Institution Staff  □ Public Servant □ Government Officer □ Professionals □ Service Personnel  □ Unemployed □ Others ( ) | | | | | | | | | |
| Personal Income per year (yuan) | □ <10,000 □ 50,000-80,000  □ 10,000-30,000 □ 80,000-120,000  □ 30,000-50,000 □ >120,000 | | | | | | | Height | | cm |
| Family Income  per year (yuan) | □ <10,000 □ 50,000-80,000  □ 10,000-30,000 □ 80,000-120,000  □ 30,000-50,000 □ >120,000 | | | | | | | Weight | | Kg |
| Education | □ No Education □ High School  □ Primary School □ University or College  □ Middle School □ Graduate School or More | | | | | | | Date of Birth | | / /  (yyyy/mm/dd) |
| Contact Person |  | | | Relationship | |  | | Tel. | |  |
| Time of Paper Reading | h/day | | | Time of screen watching | | h/day | | Time of physical exercises | | h/day |
| Smoking | years, /day | | | | | Alcohol Drinking | | years, /day | | |
| Dietary Habit | Meat or Eggs | | | □ Yes □ No | | Attitude to Survey | | □ Very satisfied □ Satisfied  □ General □ Not satisfied  □ Very unsatisfied | | |
|  | Vegetables | | | □ Yes □ No | |  |  |  |  |  |

**Part 1B. General Information (For Adolescents)**

| Name |  | | Gender | | | □ Male □ Female | | | Age |  |
| --- | --- | --- | --- | --- | --- | --- | --- | --- | --- | --- |
| Contact Person | Father: Name: Tel.:  Mother: Name: Tel.: | | | | | | | | Ethnicity | □ Han  □ Hui  □ Manchu  □ Others |
| Number of Children |  | Number of Family Members | | |  | | Medical Insurance | | □ Yes  □ No | |
| Place of Residence | Province City (County) | | | | □ Registered Rural Residents □ Registered Urban Residents | | | | | |
| School (including Grade&Class) |  | | | | | | | | | |
| Personal Income per year (yuan) | □ <10,000 □ 50,000-80,000  □ 10,000-30,000 □ 80,000-120,000  □ 30,000-50,000 □ >120,000 | | | | | | | Height | | cm |
| Family Income  per year (yuan) | □ <10,000 □ 50,000-80,000  □ 10,000-30,000 □ 80,000-120,000  □ 30,000-50,000 □ >120,000 | | | | | | | Weight | | Kg |
| Education | □ No Education □ High School  □ Primary School □ University or College  □ Middle School □ Graduate School or More | | | | | | | Date of Birth | | / /  (yyyy/mm/dd) |
| Time of Paper Reading | h/day | | | Time of screen watching | | h/day | | Time of physical exercises | | h/day |
| Smoking | years, /day | | | | | Alcohol Drinking | | years, /day | | |
| Dietary Habit | Meat or Eggs | | | □ Yes □ No | | Attitude to Survey | | □ Very satisfied □ Satisfied  □ General □ Not satisfied  □ Very unsatisfied | | |
|  | Vegetables | | | □ Yes □ No | |  |  |  |  |  |

**Part 2. Medical History**

| General Medical History | □ Hypertension For years | □ Diabetes For years |
| --- | --- | --- |
|  | □ Cardiopathy For years | □ Hyperlipemia For years |
|  | □ Cerebral infarction For years | □ Anxiety/DepressionFor years |
|  | □ Trauma For years  Injured area | □ Surgery For years  Type of surgery |
|  | □ Was a premature baby | □ Others For years  Type of disease |
|  | □ Preterm birth (Ask female) |  |
| Ophthalmic History | □ Glaucoma For years  □ Treated  □ Drugs：  □ Pilocarpine  □ Timolol  □ Cattelor / Michelangelo  □ Bromonidine / afagen  □ Brinzolamide / pyrimin  □ Prostaglandins  □ Others  □ Laser:  □ Iridectomy  □ Iridoplasty  □ LTP  □ Others  □ Operation:  □ Trabeculectomy  □ LPI  □ Others | □ Cataract For years  □ Surgery  (□ Oculus Sinister □ Oculus Dexter □ Oculus Uterque) |
|  |  | □ Diabetic retinopathy For years |
|  |  | □Retinal vein obstruction For years |
|  |  | □Macular degeneration For years |
|  |  | □ Myopia For years  Spectacles: Oculus Sinister  Oculus Dexter |
|  |  | □ Others For years  Type of disease |
| Family History | Glaucoma □ Yes □ No Family members | |
|  | Other eye diseases □ Yes □ No Family members Eye disease | |

**Part 3. Ophthalmic Examination**

Blood Pressure： / mmHg

|  | | Right Eye | Left Eye |
| --- | --- | --- | --- |
| Uncorrected visual acuity | |  |  |
| Corrected visual acuity | |  |  |
| Intraocular pressure | | mmHg | mmHg |
| Slit-lamp examination | Eyelid | □ Normal  □ Entropion  □ Ectropion  □ Trichiasis  □ Others ( ) | □ Normal  □ Entropion  □ Ectropion  □ Trichiasis  □ Others ( ) |
|  | Conjunctiva | □ Normal  □ Congestion  □ Pterygium  □ Others ( ) | □ Normal  □ Congestion  □ Pterygium  □ Others ( ) |
|  | Corneal | □ Normal  □ Edema  □ Scar  □ Ulcer  □ Others ( ) | □ Normal  □ Edema  □ Scar  □ Ulcer  □ Others ( ) |
|  | Anterior chamber depth | Center CT; Periphery CT | Center CT; Periphery CT |
|  | Aqueous humor | □ Normal □ Abnormal ( ) | □ Normal □ Abnormal ( ) |
|  | Iris | □ Normal  □ Atrophy  □ Iridotomy  □ Laser hole  □ Neovascularization  □ Others ( ) | □ Normal  □ Atrophy  □ Iridotomy  □ Laser hole  □ Neovascularization  □ Others ( ) |
|  | Pupil | □ Normal  □ Abnormal  □ Direct reflection of light | □ Normal  □ Abnormal  □ Direct reflection of light |
|  | Lens | □ Transparency  □ Turbidity  □ Intraocular lens  □ Aphakia | □ Transparency  □ Turbidity  □ Intraocular lens  □ Aphakia |
| Fundus | Optic disc | □ Boundary  □ Clear  □ Unclear / Edema  □ Color  □ Normal  □ Light  □ Hyperemia  C / D= | □ Boundary  □ Clear  □ Unclear / Edema  □ Color  □ Normal  □ Light  □ Hyperemia  C / D= |
|  | Retina | □ Normal  □ Hemorrhage  □ Exudation  □ Drusen  □ others | □ Normal  □ Hemorrhage  □ Exudation  □ Drusen  □ others |
|  | Vessel | A:  V: | A:  V: |
|  | Mac |  |  |
| Diagnosis | | □ Glaucoma  □ AACG  □ CACG  □ Suspected PACG  □ POAG  □ OHx  □ NTG  □ Suspected POAG  □ SG  □ CG  □ Cataract  □ IOL  □ Aphakia  □ DR  □ AMD  □ BRVO, BRAO  □ Ametropia  □ Xerophthalmia  □ Trachoma  □ Pterygium  □ Others | □ Glaucoma  □ AACG  □ CACG  □ Suspected PACG  □ POAG  □ OHx  □ NTG  □ Suspected POAG  □ SG  □ CG  □ Cataract  □ IOL  □ Aphakia  □ DR  □ AMD  □ BRVO, BRAO  □ Ametropia  □ Xerophthalmia  □ Trachoma  □ Pterygium  □ Others |

**Part 4. Further Examination for Cases**

Code： Name： Gender： Age：

|  | | Right eye | | Left eye | |
| --- | --- | --- | --- | --- | --- |
| Axial length (mm) | |  | |  | |
| IOP | Front |  | |  | |
|  | Behind |  | |  | |
|  | D-value |  | |  | |
|  |  | Positive / Negative / Susp | | Positive / Negative / Susp | |
| UBM | CACD |  | |  | |
|  | PPP |  | |  | |
| CCT |  |  | |  | |
| Fundus photography | RNFLD | Positive Negative | | Positive Negative | |
|  | C/D |  | |  | |
| CVF | MD |  | |  | |
|  | LV |  | |  | |
| OCT | RNFLD | Positive Negative | | Positive Negative | |
|  | C/D |  | |  | |
| Gonioscope | Upper | Static | Dynamic | Static | Dynamic |
|  | Below |  |  |  |  |
|  | Nasal side |  |  |  |  |
|  | temporal |  |  |  |  |

**References:**

1. Kleibergen F, Paap R. Generalized reduced rank tests using the singular value decomposition. Journal of Econometrics. 2006; 133(1):97-126. <https://doi.org/10.1016/j.jeconom.2005.02.011>.
2. Stock J, Yogo M. Testing for weak instruments in linear IV regression. In: Andrew D, Stock J. Identification and inference for econometric models: Essays in honor of Thomas Rothenberg. Cambridge: Cambridge University Press; 2005. P. 80-108.
3. Cragg JG, Donald S. Testing identifiability and specification in instrumental variable models. Econometric Theory. 1993; 9(2): 222-40. <https://doi.org/10.1017/S0266466600007519>.
4. Hausman J. Specification tests in econometrics. Econometrica. 1978; 46(6):1251-71.
5. Pagan AR, Hall AD. Diagnostic tests as residual analysis. Econometric Reviews. 1983; 2:159–218.
6. Durbin J. Errors in variables. Review of International Statistical Institute. 1954; 22(1/3):23-32. <https://doi.org/10.2307/1401917>.
7. Wu D. Alternative tests of independence between stochastic regressors and disturbances. Econometrics. 1974; 42(3):733-75. <https://doi.org/10.2307/1911789>
8. Wooldridge JM. Quasi-maximum likelihood estimation and testing for nonlinear models with endogenous explanatory variables. J Econom. 2014;182(1):226–34.
9. Wooldridge JM. Econometric analysis of cross section and panel data, 2nd edtion. Cambridge, MA: MIT Press; 2010.
